# Chronic Cough With Reduced Diffusing Capacity and Preserved-Ratio Impaired Spirometry in a Young Never-Smoker With Systemic Lupus Erythematosus: A Diagnostic Pitfall

**DOI:** 10.7759/cureus.114283

**Published:** 2026-08-10

**Authors:** Nivedita Singh, Divya Makkar, Govind Kumar, Vachan K Vanjarapu, Soujanya Sodavarapu

**Affiliations:** 1 Internal Medicine, Independent Physician, Motihari, IND; 2 Immunology and Rheumatology, Stanford Health Care, Pleasanton, USA; 3 Internal Medicine, San Joaquin General Hospital, Stockton, USA; 4 Dermatology, Narsingh Derma Clinic, Patna, IND; 5 Biology, Emerald High School, Dublin, USA

**Keywords:** chronic cough, diagnostic pitfall, lupus nephritis, never-smoker, preserved-ratio impaired spirometry, reduced diffusing capacity, small airway disease, systemic lupus erythematosus

## Abstract

Chronic cough in patients with systemic lupus erythematosus can be difficult to evaluate, especially when initial testing is unrevealing. We report the case of a 33-year-old woman with long-standing systemic lupus erythematosus, biopsy-proven lupus nephritis, prior extrapulmonary pericardial tuberculosis, and no active or passive smoking exposure who developed a persistent dry cough for six months. She was initially treated for gastroesophageal reflux disease with pantoprazole, without meaningful relief. Chest radiography and swallowing assessment were normal. Initial pulmonary function testing in July 2023 was formally interpreted as moderate obstructive airways disease, mild parenchymal restriction, and a moderately severe diffusion defect, without a significant bronchodilator response. High-resolution volumetric computed tomography of the chest with dynamic airway assessment showed no emphysema, bronchiectasis, interstitial lung disease, tracheomalacia, or acute cardiopulmonary process. Her cough improved substantially after inhaled therapy with albuterol and budesonide/glycopyrrolate/formoterol fumarate. Repeat pulmonary function testing in October 2023 showed improved spirometric values, but the forced expiratory volume in one second/forced vital capacity ratio remained preserved, and hemoglobin-adjusted diffusing capacity remained reduced at 64% predicted. This case highlights a diagnostic pitfall: inhaler-responsive chronic cough and a pulmonary function report suggesting obstruction should be evaluated together with post-bronchodilator spirometry, diffusing capacity, imaging, and exposure history. In systemic lupus erythematosus, a persistent cough may be associated with clinically relevant pulmonary function abnormalities even when chest imaging is unrevealing.

## Introduction

Chronic cough, generally defined in adults as a cough lasting longer than eight weeks, is a frequent symptom in clinical practice and is commonly attributed to gastroesophageal reflux disease, asthma, upper airway cough syndrome, infection, or medication-related causes. When a cough persists despite empiric treatment and chest radiography is unrevealing, pulmonary function testing becomes important for identifying physiologic abnormalities that may not be apparent on imaging [1]. Chronic obstructive pulmonary disease is usually associated with cigarette smoking, biomass exposure, occupational inhalational exposure, or advanced age. In a young never-smoker, this diagnosis should be made cautiously and supported by appropriate spirometric criteria. Current diagnostic standards require persistent post-bronchodilator airflow obstruction, generally defined by a reduced forced expiratory volume in one second (FEV1)/forced vital capacity (FVC) ratio [2,3].

Preserved-ratio impaired spirometry is a pattern in which FEV1 is reduced while the FEV1/FVC ratio remains preserved. This pattern may be symptomatic and may be mistaken clinically for obstructive airway disease, but it does not meet the classic spirometric criteria for chronic obstructive pulmonary disease [4,5]. Reduced diffusing capacity adds another layer of complexity because it may suggest abnormalities involving the alveolar-capillary interface, pulmonary vasculature, accessible alveolar volume (VA), or early parenchymal disease [3,6].

Systemic lupus erythematosus is associated with a broad range of pulmonary manifestations, including pleuritis, interstitial lung disease, pulmonary hypertension, pulmonary embolism, shrinking lung syndrome, diffuse alveolar hemorrhage, infection, and, less commonly, airway or bronchiolar involvement [7,8]. Reduced diffusing capacity has been described in nonsmoking patients with systemic lupus erythematosus, sometimes as an isolated or subclinical pulmonary-function abnormality [9,10]. We report a young lifelong never-smoker with long-standing systemic lupus erythematosus who presented with chronic cough, normal chest imaging, and concern for obstructive airway disease, but whose serial pulmonary function testing demonstrated preserved-ratio impaired spirometry with reduced diffusing capacity. This case highlights how preserved-ratio impaired spirometry with reduced diffusing capacity may mimic obstructive airway disease in systemic lupus erythematosus and lead to diagnostic uncertainty.

## Case presentation

A 33-year-old woman with systemic lupus erythematosus diagnosed in 2006 presented with a persistent dry cough lasting approximately six months. The cough was initially attributed to gastroesophageal reflux disease, and she was treated with pantoprazole. Her symptoms did not improve. She did not report fever, sputum production, hemoptysis, constitutional symptoms, or symptoms suggestive of acute lower respiratory infection.

**Figure 1:**
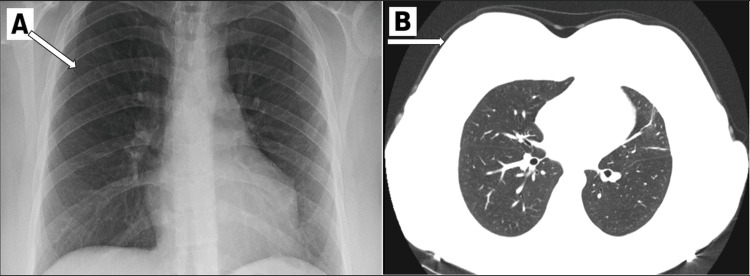
Chest radiography and computed tomography imaging. (A) Frontal chest radiograph showing no focal consolidation or acute cardiopulmonary abnormality. (B) Axial inspiratory lung-window computed tomography image showing clear lung parenchyma without emphysema, bronchiectasis, consolidation, focal nodularity, or interstitial abnormality. Dynamic airway assessment in the full study showed no evidence of tracheomalacia.

Initial evaluation included chest radiography, which was unremarkable and showed no pneumonia or other acute cardiopulmonary abnormality (Figure 1, Panel A). A swallowing assessment was also performed and was normal. Despite unrevealing initial investigations and treatment with pantoprazole, the cough persisted, prompting referral for pulmonary evaluation.

She was a lifelong never-smoker and denied passive smoke exposure from family members, friends, or close contacts. She also denied vaping, biomass fuel exposure, and relevant occupational inhalational exposure. Her weight was 87 kg, and her height was 5 feet 7 inches, corresponding to a body mass index of approximately 30.1 kg/m². Resting oxygen saturation was 98% on room air. Chest examination revealed no wheezes, crackles, or pleural rub. She was not taking an angiotensin-converting enzyme inhibitor or any other medication commonly associated with chronic dry cough.

Her systemic lupus erythematosus history was notable for positive antinuclear antibody and anti-double-stranded DNA antibody results. Renal biopsy at the time of diagnosis showed class III lupus nephritis, and she was started on azathioprine. Over the following years, she was predominantly maintained on mycophenolate mofetil, with intermittent use of azathioprine. A repeat renal biopsy in 2016 showed progression to class IV lupus nephritis. At the time of respiratory symptom onset, she was taking hydroxychloroquine 200 mg twice daily and mycophenolate mofetil. She was not receiving systemic corticosteroid therapy when the cough began.

Her history was also notable for pericardial tuberculosis in 2016, complicated by cardiac tamponade. She underwent pericardiocentesis followed by pericardial window surgery. At that time, sputum evaluation and chest imaging did not show pulmonary tuberculosis, and the disease was considered extrapulmonary, limited to the pericardium.

Because of the reduced diffusing capacity and prior pericardial disease, echocardiographic findings were reviewed. A transthoracic echocardiogram performed in June 2022, before the onset of chronic cough, showed normal left ventricular size and systolic function, with an estimated left ventricular ejection fraction of 68%. Diastolic function was normal. The right ventricle was mildly dilated but had normal systolic function. The estimated right ventricular systolic pressure was at least 15.3 mmHg, with an estimated right atrial pressure of 3 mmHg. The pulmonary artery was normal, and there was no pericardial effusion. A follow-up transthoracic echocardiogram in 2025 showed preserved biventricular function, an estimated right ventricular systolic pressure of 19.4 mmHg, and no pericardial effusion.

Initial pulmonary function testing was performed in July 2023. The report documented good patient effort and cooperation, although the patient had a significant cough during testing, particularly during the FVC maneuver. The study showed reduced FVC, FEV1, forced expiratory flow 25%-75%, total lung capacity, and diffusing capacity. There was no significant bronchodilator response. The formal pulmonary function test report interpretation was moderate obstructive airways disease, mild parenchymal restriction, and a moderately severe diffusion defect.

High-resolution volumetric computed tomography (CT) of the chest with dynamic airway assessment was performed in August 2023. The report described no change in tracheal morphology on inspiration and expiration. There was no consolidation, focal pulmonary nodule, or pleural abnormality. The overall impression was an unremarkable dynamic airway CT of the chest, with no evidence of tracheomalacia and no acute cardiopulmonary process. No emphysema, bronchiectasis, or interstitial lung disease was reported (Figure 1, Panel B).

Following the CT evaluation, the patient was started on inhaled albuterol as needed and budesonide/glycopyrrolate/formoterol fumarate twice daily. She reported noticeable improvement in her cough within two to three days of initiating inhaled therapy, with complete resolution of symptoms over the following three to four weeks. Repeat pulmonary function testing performed in October 2023 demonstrated improvement in spirometric parameters, although the post-bronchodilator FEV1/FVC ratio remained preserved, and hemoglobin-adjusted diffusing capacity of the lungs for carbon monoxide (DLCO) remained reduced (Table 1). At approximately two years of follow-up, the patient reported that when similar respiratory symptoms recurred, she resumed both prescribed inhalers (albuterol and budesonide/glycopyrrolate/formoterol fumarate) and again experienced complete symptom resolution.

**Table 1:** Serial pulmonary function testing in July and October 2023. Arrows indicate pre-bronchodilator to post-bronchodilator values where applicable. Percentages are percent predicted unless otherwise specified. DLCO = diffusing capacity of the lungs for carbon monoxide; FEF25-75 = forced expiratory flow between 25% and 75% of forced vital capacity; FEV1 = forced expiratory volume in one second; FVC = forced vital capacity; PFT = pulmonary function test; RV = residual volume; TLC = total lung capacity; VA = alveolar volume

Parameter	July 2023 initial PFT	October 2023 repeat PFT
FVC	2.43 L (65%) -> 2.64 L (70%)	2.69 L (72%) -> 2.81 L (75%)
FEV1	1.98 L (62%) -> 2.18 L (69%)	2.29 L (72%) -> 2.45 L (77%)
FEV1/FVC	82% -> 83%	85% -> 87%
FEF25-75	2.05 L/second (59%) -> 2.69 L/second (78%)	2.69 L/second (78%) -> 3.35 L/second (97%)
TLC	4.27 L (79%)	4.43 L (82%)
RV/TLC	43% (152%)	35% (123%)
DLCO uncorrected	13.40 mL/minute/mmHg (55%)	14.26 mL/minute/mmHg (59%)
Hemoglobin-adjusted DLCO	13.40 mL/minute/mmHg (61%)	14.26 mL/minute/mmHg (64%)
DLCO/VA	3.81 mL/minute/mmHg/L (85%)	4.21 mL/minute/mmHg/L (94%)
VA	3.52 L (65%)	3.39 L (63%)
Hemoglobin	10.6 g/dL	11.1 g/dL

## Discussion

The initial pulmonary function test was performed during active symptoms and was formally interpreted as moderate obstructive airways disease, mild parenchymal restriction, and a moderately severe diffusion defect. Although this interpretation and the improvement with inhaled therapy supported an airway-directed treatment trial, the post-bronchodilator FEV1/FVC ratio remained preserved in both studies, at 83% in July 2023 and 87% in October 2023. Therefore, the available spirometry did not establish chronic obstructive pulmonary disease by standard post-bronchodilator criteria [2,3] and was more consistent with preserved-ratio impaired spirometry [4,5]. Similarly, although the initial formal report used the term “mild parenchymal restriction,” this was not interpreted as definitive restrictive lung disease because total lung capacity was only mildly reduced or near the lower limit of normal and CT did not show interstitial lung disease.

The reduced diffusing capacity was the most persistent reported abnormality. Hemoglobin-adjusted DLCO was 61% predicted in July 2023 and remained reduced at 64% predicted in October 2023. DLCO/VA was relatively preserved at 85% and 94% predicted, whereas VA remained reduced at 65% and 63% predicted. Thus, reduced measured VA may have contributed to the low DLCO, although pulmonary vascular disease or early parenchymal disease could not be completely excluded [3,6]. CT showed no emphysema or interstitial lung disease.

Body habitus may have contributed to the reduced FVC because the patient’s body mass index was approximately 30.1 kg/m². Obesity can lower expiratory reserve volume and reduce measured lung volumes [11], potentially contributing to a reduced FVC with a preserved FEV1/FVC ratio [5]. However, body habitus alone does not fully explain the persistent moderate reduction in diffusing capacity, particularly in the setting of systemic lupus erythematosus.

Systemic lupus erythematosus can involve the lungs and pulmonary vasculature in several ways. Pleural disease, interstitial lung disease, pulmonary hypertension, pulmonary embolism, shrinking lung syndrome, diffuse alveolar hemorrhage, infection, and medication-related lung toxicity are usually considered first [7,8]. Reduced diffusing capacity has been described in nonsmoking patients with systemic lupus erythematosus, sometimes as an isolated or subclinical pulmonary-function abnormality [9,10].

Small airway and bronchiolar involvement may also occur in systemic autoimmune rheumatic diseases and may present with cough, dyspnea, air trapping, or abnormal pulmonary function testing before structural abnormalities are obvious on routine imaging [12].

Reduced diffusing capacity in systemic lupus erythematosus can raise concern for pulmonary vascular disease or pulmonary hypertension. Serial echocardiography did not show overt pulmonary hypertension, recurrent pericardial effusion, or ventricular dysfunction as the primary explanation. However, mild pulmonary vascular disease could not be completely excluded.

Although significant bronchodilator reversibility was not demonstrated, cough-variant asthma could not be completely excluded because bronchial provocation testing was not performed [1]. Chronic obstructive pulmonary disease was uncertain because the patient was young, had no active or passive smoking exposure, had no biomass or occupational exposure, had no emphysema on CT, had no elevation in total lung capacity, and had a preserved post-bronchodilator ratio. Although population-based data have reported an increased risk of chronic obstructive pulmonary disease among patients with systemic lupus erythematosus, that epidemiologic association does not establish the diagnosis in an individual patient without persistent post-bronchodilator airflow obstruction [13]. Post-tubercular lung disease was also less likely because the previous tuberculosis was extrapulmonary and pericardial, with no documented pulmonary involvement at that time, and the current CT did not show bronchiectasis, fibrosis, cavitation, or other structural sequelae of pulmonary tuberculosis.

Alpha-1 antitrypsin deficiency remains an important consideration in any young patient suspected of having chronic obstructive pulmonary disease or emphysema without smoking exposure. Testing is recommended in patients diagnosed with chronic obstructive pulmonary disease [14]. In this case, alpha-1 antitrypsin testing was not performed; however, the preserved ratio and absence of emphysema make classic alpha-1 antitrypsin deficiency-related emphysema less likely.

Shrinking lung syndrome is another lupus-related diagnosis worth considering. It can present with dyspnea, reduced lung volumes, and relatively normal lung parenchyma on imaging [15]. However, this patient’s dominant symptom was cough rather than dyspnea, and the available testing did not show severe restriction or a reported diaphragmatic abnormality. Therefore, shrinking lung syndrome was not established by the available data.

The response to albuterol and budesonide/glycopyrrolate/formoterol fumarate was clinically meaningful. It suggests that an airway-responsive component may have contributed to symptoms, even though spirometry did not show classic reversible obstruction. Symptomatic improvement with inhaled therapy should therefore be interpreted as a treatment response, not as proof of chronic obstructive pulmonary disease.

Taken together, the imaging findings, physiology, exposure history, and treatment response supported a cautious interpretation: an inhaler-responsive respiratory syndrome with preserved-ratio impaired spirometry and reduced diffusing capacity, rather than a straightforward diagnosis of chronic obstructive pulmonary disease.

## Conclusions

Chronic cough in a young never-smoker with systemic lupus erythematosus requires careful interpretation when symptoms, imaging, and pulmonary function testing do not point toward a single diagnosis. In this case, serial pulmonary function testing showed preserved-ratio impaired spirometry with reduced diffusing capacity despite unrevealing chest imaging. The formal initial interpretation and meaningful response to inhaled therapy supported an airway-directed treatment approach, but the preserved post-bronchodilator FEV1/FVC ratio argued against a straightforward diagnosis of chronic obstructive pulmonary disease. The main clinical lesson is that a persistent cough in systemic lupus erythematosus may warrant timely pulmonary function testing, including lung volumes and diffusing capacity, when symptoms do not improve with initial empiric therapy and early imaging is unrevealing. This approach may identify clinically relevant physiologic abnormalities earlier and help maintain a broad differential diagnosis, including autoimmune-related airway disease, pulmonary vascular disease, early parenchymal disease, shrinking lung syndrome, alpha-1 antitrypsin deficiency, and other non-smoking-related causes of chronic respiratory symptoms.
